# Designing Care Beyond the Hospital: Revealing Hidden Care Demands in Hospital-At-Home Services

**DOI:** 10.1177/00469580261466643

**Published:** 2026-07-03

**Authors:** Ranvir S. Rai

**Affiliations:** 1 Department of Leadership and Innovation, 121326 Kristiania University of Applied Sciences, Oslo, Norway

**Keywords:** hospital-at-home, digital health, health literacy, caregivers, service design, qualitative research

## Abstract

**Background:**

Hospital-at-home (HaH) services are increasingly implemented as digitally supported alternatives to inpatient care. While these models promise efficiency, continuity, and patient-centredness, they also redistribute responsibility from healthcare organisations to patients and family caregivers. This shift introduces health literacy demands related to timing, role clarity, and digitally mediated decision-making, which remain underexplored in digital health research.

**Methods:**

This qualitative study draws on ten in-depth interviews with patients, family caregivers, and healthcare professionals involved in hospital-at-home services in Norway. Interviews examined experiences of decision-making, remote monitoring, coordination, and follow-up in digitally supported home-based care. Following inductive thematic analysis, a service blueprint was developed as an analytical synthesis to examine how care demands and responsibilities were distributed across the service trajectory.

**Results:**

Caregiver burden was shaped less by individual capability than by service configurations that assumed high levels of availability, confidence, and digital competence. Family caregivers frequently assumed quasi-clinical and coordinative roles without adequate preparation or support. Health literacy demands clustered at key transitions, including decision-making, at-home monitoring, and discharge, where digital tools mediated responsibility and risk. These demands accumulated over time, generating vulnerabilities related to equity and sustainability.

**Conclusion:**

Digitally supported HaH services rely on implicit assumptions about patients’ and caregivers’ capacity to manage care at home. Design-informed analysis can reveal transition-located health literacy demands and digitally mediated responsibility shifts, supporting more inclusive and resilient digital health service models.

## 1. Introduction

Hospital-at-home (HaH) models are increasingly promoted as safe and person-centred alternatives to conventional inpatient care. They offer clinical treatment in patients’ own homes and are positioned as a way to reduce hospital stays, improve patient satisfaction, and optimise the use of healthcare resources.^1,2^ At first glance, HaH may appear to be a win–win solution, combining reduced institutionalisation with greater comfort and efficiency. However, as these models are implemented in practice, the seemingly simple idea of “care at home” reveals deeper organisational, technological, and relational complexities.

In practice, hospital-at-home services rely on digital infrastructures such as remote monitoring of vital signs, app-based symptom reporting, and asynchronous messaging to coordinate care between patients, family caregivers, and healthcare professionals outside institutional settings.^1^ In this setting, the promise of HaH is accompanied by a substantial transfer of responsibility from healthcare institutions to patients and their families. Family caregivers often take on tasks such as medication administration, symptom monitoring, coordination with health services, and digital follow-up.^3,4^ While such involvement can offer meaning and a sense of participation, it can also lead to emotional strain, uncertainty, and increased burden, particularly when caregivers are expected to manage clinical and logistical responsibilities without sufficient preparation or ongoing support.^5,6^

These shifts bring questions of health literacy into focus. Health literacy is commonly defined as a person’s ability to access, understand, appraise, and apply health information in ways that promote health and well-being.^7-9^ However, an expanding body of research emphasises that health literacy is not solely an individual capability. It is also shaped by how information is communicated, how technologies are configured, and how services are organised to support or hinder understanding and action. Limited health literacy has been associated with poorer health outcomes, increased caregiver strain, and reduced opportunities for shared decision-making.^5,10^

Digital health research increasingly recognises that literacy and competence emerge through situated interactions between users, technologies, and care environments, rather than residing solely within individuals.^11^ In digitally supported hospital-at-home services, patients and caregivers must navigate remote monitoring systems, digital communication platforms, and shifting responsibilities under conditions that are often emotionally and practically demanding. Recent studies suggest that these digitally mediated care arrangements generate uneven demands related to responsiveness, interpretation, and coordination, which are frequently absorbed by family caregivers.^12^ From this perspective, challenges in hospital-at-home services cannot be understood solely through individual capability, but must also be examined as outcomes of service configurations that embed assumptions about availability, confidence, and digital competence into everyday care practices.

This study draws on service design and co-production perspectives, which conceptualise healthcare services as relational and co-created through interactions between patients, caregivers, professionals, and technologies.^13,14^ In hospital-at-home services, patients and family caregivers do not merely receive care, but actively contribute to the delivery and coordination of care through monitoring, communication, and decision-making activities that increasingly rely on digital infrastructures. While such participation may enhance flexibility and continuity, previous research suggests that co-productive care models may also obscure inequalities in digital competence, confidence, and situational capacity if services are not intentionally designed to support diverse users.^15^

To examine how these demands emerge across the hospital-at-home trajectory, this study uses service blueprinting as an analytical lens. Originally developed as a method for visualising relationships between user actions, service encounters, and organisational processes, blueprinting has increasingly been applied in healthcare to reveal hidden coordination work, communication gaps, and structural frictions across care pathways.^16,17^ In this study, blueprinting functions not as a participatory design output, but as a design-informed analytical synthesis that integrates interview findings across patients, caregivers, and healthcare professionals. By tracing how responsibility, information, and support are distributed across transitions in care, the approach helps make visible how digitally mediated hospital-at-home services generate uneven care demands and hidden burdens beyond institutional settings.

Rather than assessing health literacy as an individual attribute, this study adopts a service design perspective to examine how care demands related to understanding, coordination, and engagement are embedded in digitally supported hospital-at-home services. Drawing on qualitative interviews and design-informed analysis, the study explores how service blueprinting can function as an analytical lens for identifying hidden care demands, structural frictions, and digitally mediated responsibility shifts across the care trajectory. By foregrounding how service design shapes everyday care practices, the study contributes to ongoing discussions on equity and sustainability in digitally supported healthcare beyond institutional settings.

## 2. Methods

This study used a qualitative, design-informed approach to examine how health literacy demands emerge within digitally supported hospital-at-home (HaH) services. The study focused on how service configurations shape responsibilities related to understanding, coordination, and digital engagement across the care trajectory.

### 2.1 Study Design

The empirical material consisted of ten anonymised in-depth interviews with patients, family caregivers, and healthcare professionals involved in hospital-at-home services in Norway. Participants were recruited through purposive sampling in collaboration with a Norwegian health organisation piloting HaH services. Sampling aimed to capture variation in caregiving roles, professional responsibilities, and interactions with digital systems rather than statistical representativeness.

The sample size was guided by the concept of information power, which suggests that smaller samples may be sufficient when participants hold information highly relevant to the study aim.^18^ The study focused on a clearly defined service context and included participants with complementary roles across the hospital-at-home trajectory. Participant characteristics are summarised in Table 1.

**Table 1.** Overview of Interview Participants

Role	Method	#
Patient	Interview	3
Family caregiver	Interview	3
Nurse (home care and hospital)	Interview	2
General practitioner	Interview	1
Service coordinator (HaH)	Interview	1
Total		10

Interviews were conducted either in person or via encrypted video conferencing tools. Each interview lasted between 45 and 75 minutes, was audio-recorded, transcribed verbatim, and anonymised. Semi-structured interview guides covered themes such as decision-making, digital follow-up, coordination across services, caregiver burden, and experiences of responsibility and support. An English translation of the interview guide is provided in Supplementary File 1.

### 2.2. Analytical Process

Interview transcripts were analysed using Braun and Clarke’s six-phase thematic analysis framework with an inductive coding approach.^19^ Coding focused on patterns related to caregiver burden, information complexity, role expectations, digital interaction, and decision-making under uncertainty.

Following thematic analysis, an analytical service blueprint was developed to synthesise recurring service structures and care demands across the hospital-at-home trajectory. The blueprint functioned as a visual analytical representation of how responsibilities, information flows, and digitally mediated interactions were distributed across service transitions. Key analytical insights derived from the blueprint are synthesised in Table 2. The full blueprint can be made available from the corresponding author upon reasonable request.

**Table 2.** Analytical Service Blueprint of Health Literacy Demands Across the Hospital-At-Home Trajectory

Analytical dimension	Before: Assessment and enrolment	During: Treatment and coordination at home	After: Follow-up and discharge
Primary service activities	Clinical assessment, eligibility decision, introduction of hospital-at-home option	Home-based treatment, remote monitoring, coordination across services	Discharge from HaH, handover to primary care, service exit
Patient role expectations	Understand care options, consent under time pressure, trust professional judgement	Monitor symptoms, interpret instructions, manage uncertainty between visits	Recognise recovery or deterioration, know when and how to seek follow-up
Caregiver role expectations	Provide availability, coordinate logistics, prepare home environment	Perform quasi-clinical tasks, manage digital tools, act as care coordinator	Resume responsibility without formal closure, manage unresolved questions
Digital touchpoints	Initial information exchange, introduction to digital tools	Apps for monitoring, communication platforms, digital documentation	Termination or continuation of digital tools without clear guidance
Health literacy demands	Rapid comprehension of unfamiliar care model, limited opportunity for dialogue	Sustained understanding of instructions, thresholds, and escalation pathways	Interpretation of discharge information and future care responsibilities
Typical friction points	Passive decision-making, unassessed caregiver capacity	Information overload, assumed digital competence, unclear responsibility	Ambiguous closure, absence of professional mandate, weak feedback loops, fragmented continuity
Equity and safety risks	Misalignment between system assumptions and user capacity	Accumulating cognitive and emotional burden, risk displacement to home	Caregiver exhaustion, delayed detection of complications
Analytical insight	Early decisions set expectations without sufficient scaffolding	Safety is co-produced through everyday navigation and improvisation	Lack of designed closure undermines sustainability and learning

### 2.3. Ethical Considerations

The study was conducted in accordance with the Declaration of Helsinki and reviewed internally by the hosting organisation’s research ethics advisor. All participants received information about the study and provided informed consent prior to participation. Interview data were anonymised upon transcription and securely stored in compliance with Norwegian data protection regulations. The study used fully anonymised qualitative material from a practice-led innovation project in a Norwegian healthcare context, consistent with national ethical guidelines for social science and humanities research.^20^

## 3. Findings

The findings illustrate how health literacy and care-related demands accumulate across the hospital-at-home (HaH) trajectory, shaping patient understanding, caregiver workload, and coordination between services. To structure the analysis, findings are presented across three phases of the HaH journey: Before, During, and After enrolment.

### 3.1. Phase 1: Before – From Illness Onset to Hospital-At-Home Assessment

This phase covers the period from illness onset to formal assessment for hospital-at-home care. Participants described this stage as marked by uncertainty, fragmented information, and informal coordination efforts, often under significant time pressure.

Across interviews, this phase emerged as a critical point where health literacy demands, system navigation, and role expectations intersected acutely.

#### 3.1.1. Patient Perspective

From the patient perspective, the assessment phase was marked by uncertainty, limited participation in decision-making, and reliance on professional judgement. Many patients reported being introduced to HaH as an option late in the process, often when they were physically exhausted or cognitively overwhelmed. As a result, their ability to engage actively in discussions about care alternatives was constrained.

Several patients described the transition from hospital to home as abrupt and poorly explained. They struggled to understand what HaH entailed, how it differed from other forms of home-based care, and what would be expected of them once at home.

“I didn’t really understand what was going on. They took some tests, and then suddenly said I could go home.” (Patient, interview)

“I had no idea what the difference was between hospital-at-home and home nursing. I just nodded along.” (Patient, interview)

Patients frequently framed their acceptance of HaH as an act of trust rather than informed choice. Trust in clinicians compensated for gaps in understanding, but also masked uncertainty about roles, risks, and responsibilities. This suggests that the digital and organisational framing of HaH limits opportunities for shared decision-making at a moment when patients’ capacity to seek clarification is reduced.

#### 3.1.2. Caregiver Perspective

For family caregivers, the “before” phase was characterised by early assumption of responsibility and extensive invisible work. Caregivers often initiated contact with healthcare services, coordinated logistics, and managed uncertainty long before any formal care plan was established. Despite this, their role was rarely acknowledged during the assessment process.

Caregivers consistently reported that no one asked about their availability, health status, work obligations, or ability to support digitally mediated care. Responsibility was implicitly assigned rather than explicitly discussed.

“No one asked if I had the capacity to be there all the time. It was just assumed.” (Caregiver, interview)

“We were left standing in the hallway, not really knowing what would happen next.” (Caregiver, interview)

Several caregivers only became aware of the full scope of their responsibilities retrospectively, once care had already shifted to the home. This delayed recognition contributed to feelings of stress, role confusion, and lack of control. The findings indicate that caregiver involvement is structurally embedded in HaH models, yet rarely formalised or supported at the point of assessment.

#### 3.1.3. Professional Perspective

Healthcare professionals described the assessment phase as shaped by clinical urgency, organisational pressure, and limited contextual knowledge. Decisions about HaH eligibility were primarily based on medical criteria, often under pressure to reduce hospital bed occupancy. At the same time, professionals acknowledged having little insight into patients’ home environments, caregiver capacity, or digital readiness.

“We assess based on medical stability, but we know very little about what the home situation is actually like.” (Nurse, interview)

“There’s pressure to free up beds, and sometimes you hope it will work out at home.” (Physician, interview)

Professionals recognised the importance of caregiver support and patient understanding but described a lack of shared tools or protocols to assess these dimensions systematically. Evaluations of caregiver capacity were often informal, based on assumptions or brief conversations, rather than structured assessment.

This reliance on intuition reflects a systemic gap: while professionals are aware that non-clinical factors matter, the service infrastructure offers limited support for integrating these considerations into decision-making. As a result, early HaH assessments may rest on partial information, increasing the risk that care demands will exceed patient and caregiver capacity later in the trajectory.

### 3.2. Phase 2: During – Treatment and Coordination at Home

This phase begins once hospital-at-home (HaH) care has been initiated and treatment is transferred from institutional to domestic settings. Participants described this phase as characterised by expanding responsibilities, reliance on digitally mediated communication, and limited opportunities to verify understanding.

While many valued the comfort and flexibility of receiving care at home, the findings also revealed how assumptions about capacity, continuity, and comprehension frequently broke down in practice.

#### 3.2.1. Patient Perspective

For patients, the treatment phase marked a transition from being primarily monitored by professionals to becoming partially responsible for their own care. Some described this shift as empowering, while others experienced uncertainty, information overload, and anxiety related to managing symptoms and treatment outside a clinical environment.

Several patients noted that professional presence in the home was intermittent, with long periods between visits and unclear channels for follow-up. This increased their reliance on memory, written instructions, or digital tools to manage care tasks.

“They came in the morning and were gone by ten. After that, it was just me sitting there, hoping I remembered everything.” (Patient, interview)

“At the hospital I could ask for help right away. At home, I didn’t want to call for every small thing.” (Patient, interview)

Patients also described challenges related to continuity. Seeing different nurses across visits required them to repeatedly explain their situation, contributing to fatigue and a sense of fragmentation.

“Sometimes it was a new nurse each day, and I had to repeat everything.” (Patient, interview)

Although being at home was often perceived as physically comforting, several patients reported feeling less secure clinically. Without immediate access to professionals, uncertainty about what constituted a “normal” symptom versus a warning sign intensified anxiety. These experiences suggest that the home setting, when insufficiently supported, can become an isolating care environment rather than a reassuring one.

#### 3.2.2. Caregiver Perspective

For family caregivers, this phase represented the peak of practical and emotional burden. Caregivers moved from supportive roles to becoming daily coordinators, symptom interpreters, and intermediaries between patients and healthcare services. Many described assuming unfamiliar tasks after minimal instruction. Digital support tools did not merely transmit information, but also required caregivers to interpret data, assess risk, and decide when escalation was necessary.

“They showed me how to give the injections once. After that, it was up to me. I was terrified.” (Caregiver, interview)

Decision-making under uncertainty was a recurring theme. Caregivers were often required to assess symptoms and decide whether to contact healthcare professionals, without clear thresholds or reassurance.

“I didn’t know how serious it was. I had to decide whether to call again, and I was afraid of overreacting.” (Caregiver, interview)

Digital tools were intended to support communication and monitoring, but several caregivers reported difficulties understanding how to use them effectively. Lack of follow-up meant that misunderstandings often went unnoticed.

“The app was supposed to help, but I didn’t really understand it. And no one checked if we were using it correctly.” (Caregiver, interview)

Importantly, caregivers described hesitating to ask questions or request clarification, fearing they would appear demanding or incapable. This reluctance contributed to suppressed uncertainty and increased emotional strain. Together, these accounts illustrate how health literacy demands are intertwined with emotional labour and power asymmetries, particularly when responsibility is high and support is perceived as limited.

#### 3.2.3. Professional Perspective

Healthcare professionals described this phase as operationally demanding and highly dependent on coordination across teams, technologies, and settings. While they valued the flexibility and patient-centred potential of HaH, they also acknowledged structural limitations in how care was organised and supported.

Professionals frequently relied on digital tools for communication and monitoring, yet had limited insight into whether patients and caregivers were able to use these tools as intended.

“We assume people use the app correctly, but we don’t really know. There’s no feedback loop.” (Nurse, interview)

Time constraints further limited opportunities to verify understanding. Short visits made it difficult to assess whether instructions had been comprehended or whether caregivers felt confident in their role.

“You have to trust that what you explained was understood. There isn’t time to go through everything again.” (Nurse, interview)

Several professionals reflected on situations where they realised, after the fact, that families were overwhelmed, despite having initially agreed to HaH.

“Sometimes I arrive and see that the family is completely exhausted. And then I realise we never really checked if they could manage this.” (Nurse, interview)

These accounts highlight a recurring pattern of assumed competence, where responsibility is transferred to the home without mechanisms for ongoing assessment or support. When health literacy is treated as a static attribute rather than a dynamic condition shaped by stress, technology, and timing, risk is effectively displaced from the healthcare system to patients and caregivers.

### 3.3. Phase 3: Follow-Up and System Exit

The final phase in the hospital-at-home (HaH) trajectory occurs when active treatment ends and patients are formally discharged from the programme. Although discharge was often framed as closure, participants frequently experienced this transition as ambiguous and weakly structured, characterised by limited feedback, unclear responsibility, and uncertainty about what would follow.

#### 3.3.1. Patient Perspective

For patients, the end of HaH care evoked mixed feelings of relief and uncertainty. While some welcomed the return to everyday routines, others felt unsure about the outcome of the treatment and about who to contact if symptoms reappeared. Several patients described the discharge as procedurally clear but experientially incomplete.

“They said I was discharged. But what does that actually mean? Should I contact my GP now, or wait?” (Patient, interview)

“When the nurse left for the last time, it felt unfinished. Like something just stopped.” (Patient, interview)

Digital tools further contributed to this ambiguity. Patients reported uncertainty about whether monitoring apps or digital reporting tools should still be used after discharge.

“I didn’t know if I was supposed to keep using the app or not. No one told me.” (Patient, interview)

These accounts suggest that discharge from HaH is often treated as a technical endpoint rather than a communicative transition. Without explicit closure or guidance, patients may be left uncertain about ongoing responsibility, increasing the risk of delayed help-seeking or disengagement from follow-up care.

#### 3.3.2. Caregiver Perspective

For family caregivers, this phase marked a release from daily caregiving demands, but also revealed delayed exhaustion and unresolved uncertainty. Several caregivers described a sudden drop in contact once HaH ended, with no structured debrief or acknowledgment of their contribution.

“When it was over, I completely crashed. I didn’t realise how exhausted I was until it stopped.” (Caregiver, interview)

“They just stopped coming. I expected someone to check in, but nothing happened.” (Caregiver, interview)

Caregivers were often unsure whether their role had formally ended or whether they were expected to continue coordinating care with primary services.

“I still had questions about medication and next steps, but I didn’t know who to ask anymore.” (Caregiver, interview)

These experiences highlight that caregiver involvement does not end neatly with clinical discharge. When services fail to provide structured handover or debriefing, caregivers may be left with unresolved concerns and emotional strain, despite having carried significant responsibility during the treatment phase.

#### 3.3.3. Professional Perspective

Healthcare professionals described the follow-up and discharge phase of hospital-at-home (HaH) services as organisationally diffuse and weakly structured. While the formal endpoint of HaH care was typically clear from a service perspective, professionals acknowledged that responsibility for ongoing monitoring and support was often assumed rather than explicitly transferred.

Several professionals described how discharge marked the end of their formal mandate, even though patients and caregivers continued to manage complex care tasks at home.

“Once they are discharged, we don’t really have a mandate anymore. And then you hope that the information has landed” (Healthcare professional, interview)

Digital infrastructures were described as offering limited support for continuity across this transition. Professionals noted that information exchange between specialist services and primary care relied on fragmented systems, reducing visibility into how patients were followed up after HaH ended.

“We use different systems. We send epikrisis, but you don’t get the same continuity of information. You don’t really see what happens afterwards” (Section leader, advanced hospital-at-home services)

Professionals also described uncertainty related to clinical responsibility when patients were no longer physically present in hospital settings. Several noted that distance, rather than clinical stability, was a key source of professional unease.

“Doctors can feel uncertainty when the patient is not in the bed, but further away” (Section leader, advanced hospital-at-home services)

Taken together, these accounts suggest that professional responsibility in the final phase of hospital-at-home care is bounded by organisational and digital infrastructures rather than by patients’ and caregivers’ ongoing needs. As a result, discharge functions as a technical endpoint rather than a designed transition, leaving residual uncertainty to be managed outside formal professional oversight.

### 3.4. Summary of Key Findings

Table 2 summarises how health literacy demands intensified at key transition points across the hospital-at-home trajectory. Across all phases, responsibility shifted towards patients and caregivers, often without corresponding adjustments in support, role clarification, or professional follow-up.

The following discussion examines the implications of these findings for the design of digitally supported hospital-at-home services, with particular attention to equity, responsibility, and sustainability.

## 4. Discussion

This study examined how health literacy demands emerge across digitally supported hospital-at-home (HaH) services and how these demands are shaped through service organisation, communication practices, and digitally mediated responsibility shifts. The findings suggest that health literacy in HaH functions not only as an individual capability, but as a systemic and relational condition produced through interactions between patients, caregivers, professionals, and digital infrastructures. Across the service trajectory, responsibility, coordination work, and decision-making demands were redistributed into domestic settings, often without corresponding adjustments in support, clarity, or preparation. The following discussion reflects on these findings through three interrelated perspectives: health literacy as a systemic condition, caregiver burden as invisible infrastructure, and implications for the design of safer and more equitable digitally supported care pathways.

### 4.1. Reframing Health Literacy as a Systemic Condition

The findings from this study suggest that health literacy in hospital-at-home (HaH) services should be understood not solely as an individual skill, but as a systemic condition shaped by how services, communication practices, and digital infrastructures are organised. Across the care trajectory, patients and caregivers were expected to interpret complex information, manage uncertainty, and navigate shifting responsibilities, often with limited opportunities to verify understanding or clarify expectations.

Rather than asking whether users are sufficiently “health literate,” the findings point towards the importance of examining how healthcare systems produce and distribute demands related to comprehension, coordination, and decision-making. This perspective aligns with research on health-literate healthcare organisations, which emphasises that services should be designed to support understanding, navigation, and participation by default.^8,10^ In this study, literacy-related challenges emerged particularly during service transitions, where information timing, role expectations, and responsibility shifts were poorly aligned.

These findings are especially relevant in digitally supported care environments, where communication and follow-up increasingly rely on remote monitoring, asynchronous messaging, and app-based coordination. While such technologies may improve continuity and flexibility, they also introduce additional interpretive and coordinative demands for patients and family caregivers. The findings suggest that these demands accumulate over time and become particularly difficult to manage when digital expectations remain implicit or insufficiently supported.

Viewed from this perspective, moments of misunderstanding, uncertainty, or delayed action should not be interpreted solely as individual failures, but as indicators of misalignment between service-generated demands and users’ situational capacity. This supports broader calls within digital health research for more socio-technical and equity-oriented approaches that recognise how care experiences are shaped through interactions between people, technologies, and organisational systems.^11,21^

### 4.2. Caregiver Burden and Digitally Mediated Responsibility

A central finding in this study is that family caregivers functioned as critical, yet largely invisible, contributors to service delivery within hospital-at-home services. Caregivers frequently assumed responsibilities that extended beyond traditional supportive roles, including symptom interpretation, coordination across services, digital monitoring, and risk assessment in everyday care situations. However, these responsibilities were rarely formalised, systematically assessed, or consistently supported by healthcare services.

Importantly, caregiver burden emerged not as isolated tasks, but as an ongoing accumulation of responsibility across the care trajectory. As professional oversight became increasingly mediated through digital communication and remote follow-up, caregivers often became responsible for translating instructions into action, interpreting changes in patient condition, and deciding when escalation was necessary. In this sense, digitally supported HaH services redistributed not only practical tasks, but also uncertainty and accountability into domestic settings.

These findings reinforce concerns raised in previous research suggesting that co-productive healthcare models may unintentionally obscure inequalities in capacity, availability, and digital competence.^12,15^ While participation and involvement are often framed positively within health service innovation, the findings from this study illustrate how participation may also become burdensome when expectations are insufficiently clarified or supported. In particular, caregivers frequently described uncertainty regarding clinical thresholds, communication pathways, and responsibility boundaries.

The findings also demonstrate how digital technologies themselves shape the distribution of responsibility. Monitoring applications, asynchronous communication systems, and remote reporting tools did not merely support communication; they implicitly structured expectations regarding who was responsible for noticing deterioration, interpreting symptoms, and initiating contact with healthcare professionals. When such expectations remained unclear, caregivers absorbed additional emotional and coordinative burdens that were largely invisible within formal service structures.

Taken together, these findings suggest that caregiving in digitally supported HaH services functions as a form of invisible infrastructure that sustains continuity and safety outside institutional settings. However, when this infrastructure remains unsupported, the redistribution of responsibility may undermine both equity and patient safety. Designing safer hospital-at-home services therefore requires explicit attention to caregiver roles, digital support needs, and the organisational conditions under which participation becomes sustainable.

### 4.3. Implications for Digitally Supported Hospital-At-Home Services

The findings from this study carry several implications for the future design and organisation of digitally supported hospital-at-home services. Most importantly, they suggest that continuity of care alone is insufficient if patients and caregivers lack clarity regarding responsibilities, expectations, and decision-making processes. Across the service trajectory, participants frequently described uncertainty not because information was entirely absent, but because information, timing, and responsibility were poorly aligned.

From a service design perspective, this highlights the importance of designing for clarity rather than assuming that continuity of contact automatically produces understanding or reassurance. The blueprint-based analysis demonstrated how confusion and uncertainty clustered around key transitions, particularly during enrolment, home-based monitoring, and discharge. These transition points generated concentrated health literacy demands where users were required to interpret information and act under conditions of uncertainty.

Several practical implications follow from these findings. First, services may benefit from more structured onboarding processes for family caregivers, including explicit clarification of roles, escalation procedures, and expectations related to digital tools and communication pathways. Second, digital interfaces and monitoring systems should be designed to externalise important decision criteria and reduce reliance on subjective interpretation under stressful conditions. Third, discharge from hospital-at-home services should be treated not only as a technical endpoint, but as a relational transition requiring closure, clarification, and follow-up.

Methodologically, the study also demonstrates how design-informed analytical tools such as service blueprinting can contribute to digital health and health services research. By tracing how information, responsibility, and support are distributed across service transitions, blueprinting helped reveal hidden care demands and structural frictions that were difficult to capture through thematic analysis alone. This supports growing interest in socio-technical and systems-oriented approaches within digital health research that move beyond narrow implementation or adoption perspectives.^21,22^

## 5. Limitations

This study has several limitations. First, the empirical material was generated within a service development context and later analysed using a design-informed qualitative approach. Although this provided access to rich real-world experiences from hospital-at-home (HaH) services, the interviews were not originally designed to test predefined research hypotheses, and some topics may therefore have been explored unevenly across participants.

Second, the study was conducted within a single Norwegian health region and included a relatively small sample of patients, family caregivers, and healthcare professionals. While qualitative studies aim for depth rather than statistical generalisation, the findings may not fully reflect organisational, cultural, or infrastructural differences across other healthcare settings or user groups.

Third, most caregivers included in the study were older spouses. The experiences of younger caregivers, multicultural families, or individuals with limited digital access were less represented and may involve different forms of health literacy and digital support challenges.

Finally, the study focused on identifying systemic care demands and structural frictions rather than evaluating specific interventions or implementation outcomes. Future research could build on these findings by testing targeted design interventions and examining how digitally supported hospital-at-home services can be adapted more safely and equitably across diverse care contexts.^23^

## 6. Conclusion

Hospital-at-home (HaH) services hold considerable promise for improving patient experience and reducing pressure on hospital systems. This study shows, however, that digitally supported HaH models also redistribute responsibility, coordination work, and decision-making demands from healthcare institutions to patients and family caregivers. Across the care trajectory, information timing, role clarity, and digitally mediated interactions shaped how responsibility and risk were negotiated in everyday care practices. When these elements were poorly aligned, cognitive and coordinative burdens were silently shifted to households, particularly to family caregivers who frequently assumed quasi-clinical and digital responsibilities without adequate preparation or support.

By adopting a design-informed analytical perspective, the study demonstrates how service blueprinting can help reveal hidden care demands and structural frictions embedded in digitally supported care pathways. Rather than treating health literacy as solely an individual capability, the findings position it as a systemic and relational condition shaped by service design, communication practices, and digital infrastructures. This contributes to ongoing discussions in digital health and health services research concerning how hospital-at-home services can be designed more safely, equitably, and sustainably beyond institutional settings.^15,21^

## Supplemental Material

Supplemental Material - Designing Care Beyond the Hospital: Revealing Hidden Care Demands in Hospital-at-Home Services Supplemental Material for Designing Care Beyond the Hospital: Revealing Hidden Care Demands in Hospital-at-Home Services by Ranvir S. Rai in INQUIRY: The Journal of Health Care Organization, Provision, and Financing.

## Data Availability

The datasets generated and analyzed during the current study are not publicly available due to privacy and confidentiality considerations but are available from the corresponding author on reasonable request.*
