# Supplementary material for: Designing Care Beyond the Hospital: Revealing Hidden Care Demands in Hospital-At-Home Services
Source: Inquiry. 2026 Jul 3;63:00469580261466643. doi: 10.1177/00469580261466643 (PMC13332287; doi:10.1177/00469580261466643)
Supplement: Supplemental Material - Designing Care Beyond the Hospital: Revealing Hidden Care Demands in Hospital-at-Home Services [file sj-pdf-1-inq-10.1177_00469580261466643.pdf]

Supplementary File 1: Interview Guides

This semi-structured interview guide was developed to explore the experiences of family caregivers and healthcare professionals involved in hospital-at-home (HaH) services in Norway.

Two versions of the guide were used: one for representatives of caregiver support organizations, and one for project leads and service managers responsible for HaH implementation. The interviews focused on perceived roles, support structures, digital and medical tools, and system-level coordination. Below is an English translation of the most relevant questions used in the study.

Guide 1: Family Caregiver Support Representatives
Used with professionals from caregiver organizations such as The Family Caregiver Centre

Background and Role

- Can you tell me about your role and your position at the Family Caregiver Centre / Psychiatric Nurse Consultant?
- Can you describe the services offered at the caregiver centre?
- How do you work with youth caregivers, caregiver programs, and training for professionals?

Caregiver Experiences

- What kinds of inquiries do you receive from caregivers?
- What are common challenges faced by caregivers? How do you assist them?
- Are there specific recurring themes or situations you observe among caregivers?

Hospital-at-Home (HaH)

- What are your thoughts on the development of hospital-at-home and home-based treatment? Strengths? Challenges?
- Do you receive questions from caregivers regarding HaH or medical care at home?
- How are caregivers involved in the decision to shift from hospital to home care?
- How is training or preparation for caregivers handled? What feedback have you received?
- How do caregivers experience the responsibility of having the patient at home vs. in hospital?
- What are the advantages or disadvantages for caregivers when patients receive care at home?
- Do you hear concerns or worries about HaH? What makes caregivers feel safe?

Digital Tools & Medical Equipment

- Can you share any experiences regarding caregivers and technical or medical equipment at home?
- How do you feel about patients using digital tools to communicate or self-manage at home? Challenges?
- Do you receive caregiver inquiries about digital health tools or platforms?

- What about medical devices at home – any recurring issues or concerns?
- What are the biggest opportunities or risks you see when it comes to technology?

Economy & the System

- Do caregivers describe financial difficulties related to the healthcare system?
- What is your view on the collaboration between primary and specialist care?
- What stands out to you regarding the economic burden on caregivers when a loved one becomes ill?

Final reflections

- Are there any other topics or challenges we haven't touched on that you think we should include?

Guide 2: HaH Project Leads and Service Managers
Used with professionals responsible for operating or developing hospital-at-home services

Experience with HaH

- Can you describe your work and role as project leader for HaH services?
- What projects or services are you currently managing?
- How long have you worked with HaH?
- Can you share insights about capacity, impact, and economics of HaH at your institution?
- What are your strategic goals for HaH? What proportion of patients are expected to receive care at home in the future?

Diagnoses and Criteria

- Are there specific diagnoses you find especially suited for HaH? Any that are not?
- How was the process of identifying appropriate conditions for HaH structured?
- Have any particular diagnoses been excluded? Why?
- How is patient eligibility assessed for HaH?

Patients & Caregivers

- What makes a patient suitable for HaH vs. hospital-based care?
- How do you assess the role and capacity of caregivers?
- What worries or challenges do you hear from caregivers?
- What preparation and support are caregivers given before home treatment begins?
- How is their consent obtained? Are they formally involved in the decision?
- What kind of training or orientation is given to staff?
- What backgrounds or roles do your HaH staff typically have?

Logistics

- How do you handle logistics such as transport, number of patients, treatment coordination, and follow-up?
- Do you have a designated coordinator role?
- What tools or systems do you use?

Communication and Equipment

- How do you communicate with patients and caregivers during HaH care?
- What tools or systems are used for this communication?
- How is medical equipment in the home managed and delivered?
- What challenges arise in replicating a hospital-like environment in the home?

Collaboration with Primary Care & Municipal Services

- You mentioned collaboration with municipal care – could you describe how that works?
- In what situations is the municipality typically involved?
- Are there recurring challenges or opportunities in this collaboration?
- How are responsibilities shared between sectors?

Policy and Regulations

- Do any policy or legal frameworks support HaH or home-based treatment?
- How do you navigate national guidelines or sector-specific standards?

Final reflections

- Are there any issues we haven't discussed that you think are important to include?
